# The Adenosinergic Signaling: A Complex but Promising Therapeutic Target for Alzheimer’s Disease

**DOI:** 10.3389/fnins.2018.00520

**Published:** 2018-08-03

**Authors:** Lucrezia Cellai, Kevin Carvalho, Emilie Faivre, Aude Deleau, Didier Vieau, Luc Buée, David Blum, Céline Mériaux, Victoria Gomez-Murcia

**Affiliations:** Institut National de la Santé et de la Recherche Médicale, CHU Lille, UMR-S 1172-JPArc, LabEx DISTALZ, Université de Lille, Lille, France

**Keywords:** Alzheimer’s disease, memory, caffeine, adenosine, adenosine receptors

## Abstract

Alzheimer’s disease (AD) is the most common neurodegenerative disorder in elderly people. AD is characterized by a progressive cognitive decline and it is neuropathologically defined by two hallmarks: extracellular deposits of aggregated β-amyloid (Aβ) peptides and intraneuronal fibrillar aggregates of hyper- and abnormally phosphorylated Tau proteins. AD results from multiple genetic and environmental risk factors. Epidemiological studies reported beneficial effects of caffeine, a non-selective adenosine receptors antagonist. In the present review, we discuss the impact of caffeine and of adenosinergic system modulation on AD, in terms of pathology and therapeutics.

## Introduction

Alzheimer’s disease (AD) is the most common form of dementia, representing 70% of cases affecting more than 40 millions patients worldwide (Scheltens et al., 2016). The anatomopathological diagnosis of AD is based on the presence of two lesions: amyloid deposits and neurofibrillary tangles. Amyloid deposits are composed of extracellular accumulation of beta amyloid peptides (Aβ) resulting from the sequential cleavage of the amyloid precursor protein (APP) by beta- and gamma-secretases (De Strooper, 2010). Neurofibrillary tangles result from intra-neuronal accumulation of hyper- and abnormally phosphorylated Tau protein (defined as “Tau pathology”). Although the development and progression of both lesions are involved in the evolution of clinical deficits, the spreading of Tau pathology has been suggested as a more reliable predictor for cognitive impairment (Brier et al., 2016). In parallel, complex neuroinflammatory and neuroimmune processes, involving innate (notably microglia, astrocytes) and adaptive (T cells, Tregs cells) brain resident or peripheral immune cells, were shown to be strongly involved in both the development of AD lesions and cognitive deficits (for reviews see Dansokho et al., 2017; Leyns and Holtzman, 2017; Ising and Heneka, 2018; Laurent et al., 2018). The role of immune system into the AD pathophysiological process is supported by the findings of several variants in immunity-related genes resulted as susceptibility markers in genome wide association studies (i.e., CR1, SPI1, the MS4As, TREM2, ABCA7, CD33, and INPP5D; Efthymiou and Goate, 2017).

**FIGURE 1:**
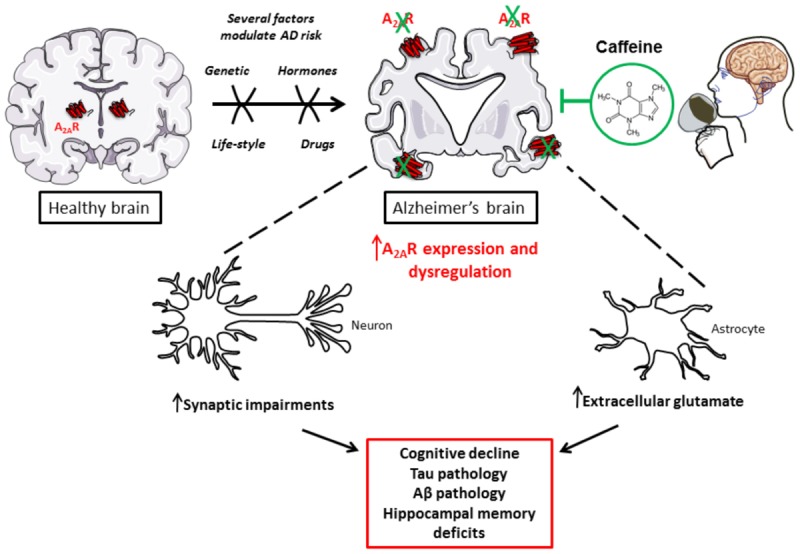
Caffeine, as a new promising treatment to counterbalance toxic impact of A_2A_R dysregulation in AD.

Alzheimer’s disease is mostly a sporadic disease (Scheltens et al., 2016); indeed, the etiological mechanisms underlying neuropathological changes in AD remain unclear so far, but they appear to be dependent on both genetic and environmental factors (Reitz et al., 2011). A recent study estimated that around 35% of dementia is attributable to a combination of modifiable lifestyle factors, some of them linked to cardio-metabolic changes (Livingston et al., 2017). Interestingly, compelling epidemiologic evidences support that habitual consumption of caffeine is prone to reduce cognitive decline with aging and to reduce AD risk (reviewed in Flaten et al., 2014; Cunha, 2016). This mini-review will be focused on the current knowledge regarding the mechanisms underlying caffeine beneficial effects in AD, and specifically on how such positive effects are ascribable to an impact of caffeine on adenosinergic signaling (**Figure 1**).

## Caffeine, Cognition, and AD

The pharmacological actions of caffeine are complex and greatly differ depending on its dosage and concentration. Its effect may also depend on its metabolites: paraxanthine (1,7-dimethylxanthine), theophylline (3,7-dimethylxanthine), and theobromine (1,3-dimethylxanthine). Pharmacokinetics and metabolism of caffeine have been extensively reviewed recently (Nehlig, 2018).

In healthy subjects, besides its positive effect on mood, alertness, attention and information processing (Sawyer et al., 1982; Fredholm et al., 1999, 2005; Smit and Rogers, 2000; Lorist and Tops, 2003; Fisone et al., 2004; Haskell et al., 2005), caffeine has also been shown to favor neuronal excitability in neocortex (Kerkhofs et al., 2017) and memory consolidation (Borota et al., 2014). Its widespread consumption and beneficial impact on cognitive functions maintenance emphasizes the need to study the effects of caffeine consumption on AD pathophysiology and aging-associated decline. In 2002, a retrospective study supported an inverse correlation between caffeine intake and age at AD onset. Indeed, AD patients presented an average daily caffeine intake of about 74 ± 98 mg during the 20 years preceding AD diagnosis, whereas age-matched controls had a larger average daily caffeine intake of 199 ± 136 mg during the corresponding 20 years of their lifetime (Maia and de Mendonça, 2002). Five years later, a 4-year-long observational study, in a group of over 7,000 participants, revealed a significantly lower deterioration in verbal retrieval and visuospatial memory in >65 years old women, who consumed over 3 cups of coffee a day as compared to women who consumed 1 cup of coffee a day or less. However, such findings were not reported in men (Ritchie et al., 2007). A longitudinal population-based study found that a daily intake of 3–5 cups of coffee in middle-aged people lower the risk of AD and dementia disease by around 65% as compared to lower amounts of coffee (Eskelinen et al., 2009). A meta-analysis of 9 cohorts and 2 case-control studies also reported an inverse correlation between AD incidence and caffeine intake (Santos et al., 2010). In contrast, the Honolulu-Asian Aging Study reported no significant correlation between midlife coffee or caffeine consumption and the risk of dementia or cognitive impairment. However, the authors reported that the higher caffeine intake (>277.5 mg per day) was associated with a decreased risk to present, at autopsy, any of the neuropathological lesions, i.e., AD-related lesions, microvascular ischemic lesions, cortical Lewy bodies, hippocampal sclerosis, or generalized atrophy (Gelber et al., 2011). A further possible positive impact of caffeine on cerebrospinal fluid (CSF) production and turnover, finally facilitating Aβ clearance, was even suggested (Wostyn et al., 2011).

In line with the latter epidemiological and neuropathological observations, compelling experimental evidence, both *in vivo* and *in vitro*, give reliable proof-of-concept that caffeine has a strong potential toward AD pathology and associated cognitive deficits. In seminal studies, Arendash et al. demonstrated that caffeine intake in transgenic mice overexpressing mutated APP (APPsw) alleviates cognitive decline induced by Aβ and lowers the concentration of this neurotoxic peptide in both preventive and therapeutic paradigms. APPsw mice chronically treated from 4 to 9.5 months of age with caffeinated water (300 mg/l, corresponding to 500 mg per day in humans), exhibited improved working and spatial memories as well as reduced levels of hippocampal Aβ_1-40_ and Aβ_1-42_. At late pathological stages (18–19 month), APPsw mice treated similarly for 4–5 weeks display reversed memory deficits and reduced Aβ deposits and soluble Aβ levels in entorhinal cortex as well as in hippocampus (Arendash et al., 2006, 2009). Furthermore, in SweAPP N2a cell cultures – murine neuron-like cells transfected with the human “Swedish” mutant APP – the treatment with different concentrations of caffeine (below 10 μM) induced a reduced production of Aβ_1-40_ and Aβ_1-42_ (Arendash et al., 2006). Molecular dynamics simulations recently suggested that the hydrophobic core-recognition motif of amyloid peptide formation could be physically blocked by caffeine, thereby abolishing the self-assembly formation (Sharma and Paul, 2016). Our previous studies also emphasize that chronic caffeine treatment prevents the development of spatial memory deficits, reduces hippocampal Tau phosphorylation and proteolytic fragments as well as mitigates parenchymal neuroinflammation in a model of AD-like Tau pathology (Laurent et al., 2014). All these data are in accordance with the decrease of Aβ production and Tau phosphorylation in rabbits fed with a high cholesterol diet – an experimental model for sporadic AD – treated with low and high doses of caffeine (0.5–30 mg per day, corresponding to a maximal 60 mg per day in humans) (Prasanthi et al., 2010). Thus, a growing body of evidence indicates that caffeine is able to reduce behavioral and pathological features associated with AD in models of sporadic and early onset (genetically-linked) AD. It is however important to mention that a recent study performed in transgenic mice developing both amyloid and Tau lesions suggests that chronic caffeine consumption may lead to adverse effect notably by enhancing BPSD (Behavioral and Psychological Symptoms of Dementia)-like symptoms that may interfere with the ability of caffeine to normalize memory deficits (Baeta-Corral et al., 2018), underlying possible side effects that need to be carefully evaluated in future trials in patients. Overall, while a large amount of epidemiological and experimental evidence implies that caffeine and associated methylxanthines may have beneficial long-term protective effects against late-life cognitive impairment or decline, clinical evaluations are still warranted. Relationships between caffeine levels and AD biomarkers remain also unclear so far (Travassos et al., 2015). Therefore, a long-term interventional randomized controlled study taking into account CSF and blood biomarkers but also possible side effects is required to definitively conclude on the therapeutic potential of caffeine in AD and dementia. Possibly, future studies will also need to evaluate whether caffeine itself or its metabolites are presumably responsible for clinical and metabolic changes. Indeed, previous data indicate that the CSF and plasma level of one of the main metabolites of caffeine, theobromine, could be associated with Aβ_42_ CSF levels suggesting that it may have a particular impact (Travassos et al., 2015).

## Caffeine, Adenosine, and Adenosine Receptors

Under normal consumption conditions, caffeine acts as a non-selective competitive antagonist of the four subtypes of G-protein-coupled adenosine P1 receptors: A_1_, A_2A_, A_2B,_ and A_3_ whose endogenous ligand is adenosine, a purinergic nucleoside. Among them, A_1_ and A_2A_ receptor subtypes are the most abundant in the mammalian brain. A_1_Rs are widely distributed throughout the central nervous system (CNS) while A_2A_Rs are mainly expressed by striato-pallidal medium spiny neurons in the striatum. However, A_2A_R is largely expressed within other area of the CNS at lower levels. A_1_R and A_2A_R are both G-protein-coupled receptor, the former being coupled to inhibitory G proteins (Gi), the latter to activatory G proteins (G_olf_ or Gs; Fredholm et al., 2005). Adenosine receptors are expressed by neurons, glial and endothelial cells. A_1_Rs particularly regulate excitatory transmission at both pre- and post-synaptic sites. In synapses, A_2A_Rs are known to fine-tune synaptic plasticity, notably by regulating presynaptic release of glutamate (Cunha, 2016). Recent data emphasized an important role of A_2A_Rs in regulating glutamate and GABA uptake by astrocytes (Matos et al., 2012a,b; Cristóvão-Ferreira et al., 2013).

**FIGURE 2:**
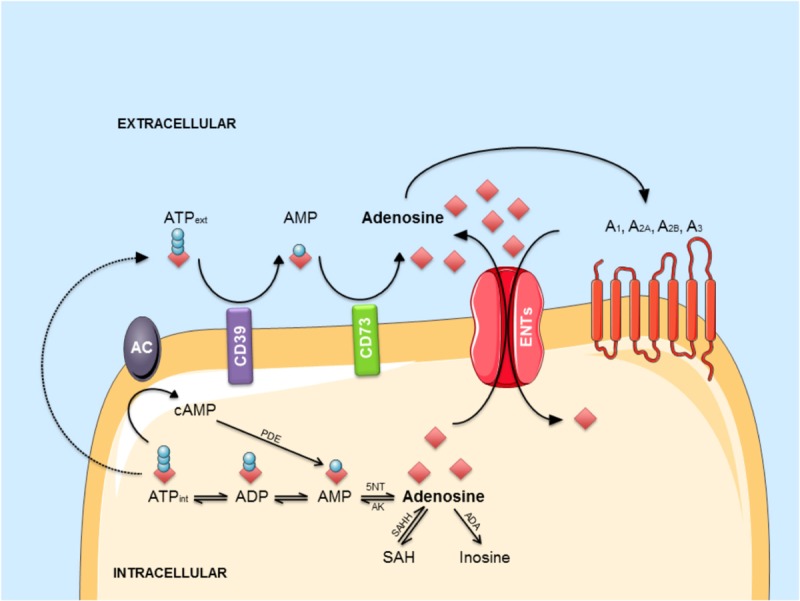
The adenosinergic pathway. Intracellular adenosine is generated from AMP or SAH. After intracellular uptake, it is phosphorylated to AMP by AK or converted into inosine by ADA. ATP is released into the extracellular environment and hydrolyzed by the concerted action of CD39 and CD73 into adenosine. In response to metabolic stress, adenosine accumulates in the extracellular environment. Extracellular adenosine can bind to four different G-protein-coupled receptors (A_1_, A_2A_, A_2B,_ and A_3_) or be transported back into the cell via ENTs. Abbreviations: A_1_, A_2A_, A_2B_, A_3_, adenosine receptors; AC, adenylyl cyclase; ADA, adenosine deaminase; AK, adenosine kinase; AMP, adenosine monophosphate; ATP, adenosine triphosphate; cAMP, cyclic AMP; ENT, equilibrative nucleoside transporter; 5NT, 5’-nucleotidase; PDE, phosphodiesterase; SAH, S-adenosyl-homocysteine; SAHH, S-adenosyl-homocysteine hydrolase.

Adenosine can be generated from adenine nucleosides present inside the cells or on the outer side of the plasma membrane (Zimmermann, 2000), via intracellular or extracellular 5′-nucleotidases enzymes (**Figure 2**). At extracellular level, several mechanisms can contribute to adenosine generation. ATP can be released by distinct mechanisms: (1) by exocytosis from both glial cells (Imura et al., 2013) or neurons, where it is released as co-transmitter and as a neurotransmitter acting on P2 purinergic receptors (Zimmermann, 1994; Burnstock, 2007); (2) by lysosomal exocytosis (Jung et al., 2013, 2014) or (3) via hemichannels (Chever et al., 2014; Lopatář et al., 2015; Orellana et al., 2015; Orellana, 2016). Cyclic adenosine 3′–5′ monophosphate (cAMP) can also be released by cells via a probenecid-sensitive transporter or converted into 5′-AMP inside the cells and then released (Rosenberg and Li, 1995; Brundege et al., 1997). Once out of the cell, these nucleotides undergo an enzymatic conversion through coupled ectonucleotidases CD39/CD73 which sequentially converts ATP/ADP to AMP and AMP to adenosine (Zimmermann, 2006; Zimmermann et al., 2012). Adenosine may be then further transformed to inosine by extracellular adenosine deaminase enzymes (ADA) or may enter inside the cell through bidirectional equilibrative nucleoside transporters, ENT1 and ENT2, where it may undergo intracellular ADA action thus generating inosine, or it may be phosphorylated to AMP by adenosine kinases (ADK; see Borea et al., 2016). In the cytosol, AMP-specific 5′-nucleotidase, mainly accounts for adenosine generation during enhanced metabolic load (Hunsucker et al., 2001) while *S*-adenosylhomocysteine (SAH) pathway plays a negligible role (see Latini and Pedata, 2001) except regarding the non-receptor mediated epigenetics effect of adenosine (Williams-Karnesky et al., 2013). Once generated, intracellularly-generated adenosine can be released via ENT1 and ENT2 in the outer space of the cell (King et al., 2006) contributing to the maintenance of a basal adenosine tone (Lee et al., 2018).

Extracellular adenosine is central in the regulation of several brain functions as it is tightly linked to the energetic state of neurons; its local increase notably reflects the depletion of ATP intracellular storage (Parkinson et al., 2011) that may be rather due to increased metabolic demand than metabolite availability. This happens in several conditions, including hypoxia, hypoglycemia and high frequency stimulation/seizures. It has also been found that adenosine extracellular concentration varies during physiological processes, where it represents a brain fatigue factor (Porkka-Heiskanen et al., 1997), thus, playing a role in sleep-wake cycle (reviewed in Murillo-Rodriguez et al., 2003; Porkka-Heiskanen and Kalinchuk, 2011; Chen, 2014; Huang et al., 2014), and cerebral blood flow (Gordon et al., 2008; McClure et al., 2011).

In neuronal cells, adenosine regulates survival and neurotransmitter release (Cunha et al., 2008). In glial cells, it is involved in the control of differentiation (Coppi et al., 2013, 2015), reactivity (Newell et al., 2015; Madeira et al., 2016; Liu et al., 2018), proliferation (George et al., 2015), and neurotransmitter uptake (Matos et al., 2012a,b, 2013; Cristóvão-Ferreira et al., 2013). The role of the physiological increased adenosine tone has been interpreted as involved in neuronal homeostasis, representing a sort of link between energy metabolism and neuronal excitability. Instead, under condition of cellular stress, adenosine tone is raised up that could eventually counteract glutamate neurotransmission (Brambilla et al., 2005). This phenomenon occurs in pathological contexts such as epilepsy (Ilie et al., 2012; Boison, 2016; Cieslak et al., 2018), ischemia or oxygen-glucose deprivation (Melani et al., 1999; zur Nedden et al., 2014), inflammation (Mukandala et al., 2016), and AD (Alonso-Andrés et al., 2018).

To which extent changes of adenosine level, recently observed in the temporal cortex of AD patients (Alonso-Andrés et al., 2018), are involved in the pathophysiological development remains an unsolved question. On the one hand, an increase in the endogenous level of adenosine in the diseased parenchyma could represent a beneficial response, at least at short-term. Indeed, first, increased adenosine tone allows a higher production of SAH, which suppresses methyltransferase activity and subsequently reduces DNA methylation (Williams-Karnesky et al., 2013). DNA methylation is an interesting aspect of the adenosine effect to be taken into account, since dynamic and complex changes in DNA methylation profiles have been reported in the brain of both mice and patients with AD (Sanchez-Mut et al., 2013; Qazi et al., 2018). Secondly, increased adenosine would be prone to favor A_1_R activation, presumably normalizing the hyperexcitability and excitotoxicity network occurring in AD parenchyma (Blum et al., 2003; Cunha, 2016). Therefore, increasing adenosine tone, for instance by blocking ENT1 transporter, could be seen as a therapeutic option in AD as nicely recently demonstrated (Lee et al., 2018). Given that chronic A_1_R activation favors activation effect and excitotoxicity occurrence due to receptor internalization (Blum et al., 2003) and that A_2A_R overactivation leads to memory impairments and AD pathology development (see below), the benefits afforded by a chronic increase of adenosine tone (Lee et al., 2018) are presumably not mediated by receptor-mediated effects. On the other hand, brain adenosine surge in AD could represent a detrimental signal fitting with the above-mentioned ability of caffeine to prevent AD pathology and related cognitive deficits. Several publications emphasize that blockade of A_2A_ (see below) but also A_1_ (Dennissen et al., 2016) and A_3_ (Li et al., 2015) receptors might be of interest in the treatment of AD.

## The Role of Adenosinergic A_2A_ Receptor in AD

Persisting higher adenosine tone is thought to preferentially lead to A_2A_R activation over A_1_R (Cunha, 2016). Several works argue for a detrimental impact of A_2A_R in AD pathophysiology, which could readily explain the beneficial effects of caffeine. In line with a central role of A_2A_Rs in AD, recently, an association between a polymorphism of the ADORA2A gene with hippocampal volume in mild cognitive impairment and AD was reported (Horgusluoglu-Moloch et al., 2017). A_2A_R expression and function have been shown abnormally enhanced in AD brain and, accordingly, blocking A_2A_R has been proven beneficial; thus, underlying the detrimental contributing impact of the enhancement of A_2A_R signaling in the pathological brain. Indeed, besides a higher adenosine tone, A_2A_R expression and function appear to be dysregulated in AD. Early studies on cortical plasma membranes revealed that cortex of AD patients exhibit enhanced A_2A_R binding and response (Albasanz et al., 2008). More recent data emphasized that A_2A_R expression is abnormally increased not only in brain cortical parenchyma of AD patients (Orr et al., 2015; Temido-Ferreira et al., 2018) but also on transgenic mouse models (Viana da Silva et al., 2016; Faivre et al., 2018; Lee et al., 2018; Orr et al., 2018; Silva et al., 2018). A_2A_R changes have been shown to occur at both neuronal/synaptic (Viana da Silva et al., 2016; Temido-Ferreira et al., 2018; Silva et al., 2018) and astroglial (Orr et al., 2015, 2018; Lee et al., 2018; Faivre et al., 2018) levels. Interestingly, the sole activation of A_2A_R induced by a brain injection of an agonist is sufficient to promote memory deficits (Pereira et al., 2005; Pagnussat et al., 2015). In addition, conditional neuronal activation of A_2A_R or related transduction pathways using optogenetics (Li et al., 2015) promotes memory and plasticity deficits in hippocampus (Giménez-Llort et al., 2007; Batalha et al., 2013, 2016; Temido-Ferreira et al., 2018). With regards to astrocytes, activating A_2A_R (Matos et al., 2013) or associated transduction pathways in astrocytes is also prone to favor the emergence of hippocampal deficits with impaired glutamate uptake, thanks to a regulation of the Na^+^/K^+^ ATPase regulating GLT-1 transporter, presumably favoring memory deficits (Matos et al., 2012a,b, 2013; Orr et al., 2015). On the other side, A_2A_R blockade or deletion was also found to counteract synaptotoxicity and memory deficits acutely induced by β-amyloid peptides (Dall’Igna et al., 2003, 2007; Canas et al., 2009). Furthermore, several works underline that blocking A_2A_R using pharmacological or genetic tools improves memory deficits and even pathology as well as parenchymal inflammation in different experimental transgenic AD models chronically developing either amyloid burden and Tau pathology (Orr et al., 2015, 2018; Laurent et al., 2016; Faivre et al., 2018; Silva et al., 2018). Even this still remains controversial (Lu et al., 2016), it is noteworthy that some data had indicated that A_2A_R might impact on the production of Aβ *in vitro* (Nagpure and Bian, 2014). Finally, this is in line with our observations that CD73 blockade, likely reducing adenosine tone and A_2A_R activity, favors the non-amyloidogenic pathway, counteracting Aβ formation. The overall view on A_2A_R as a therapeutic target thus looks promising.

It is notable that A_2A_R antagonism has been reported as a promising approach in Parkinson’s Disease prone to counteract both motor, cognitive symptoms and α-synproteinopathy (Kachroo and Schwarzschild, 2012; Ferreira et al., 2016, 2018). While some clinical trials failed to demonstrate a large benefit of A_2A_R blockade as monotherapy in PD patients even reducing off-time (SYN-115; Hauser et al., 2014), the A_2A_R antagonist istradefylline (KW-6002) is still tested in phase III and it is already approved as therapeutic option for PD patients in Japan where it is early administered in association with L-DOPA to reduce motor symptoms and dyskinesia (Borea et al., 2016; Oertel and Schulz, 2016). Considering safety and tolerability of A_2A_R antagonists in PD trials, it is tempting to consider that a repurposing of these molecules could be of great interest in AD considering their ability to normalize cognitive and to reduce several pathophysiological mechanisms, from synaptic deficits to lesion development passing through neuroinflammation.

## Conclusion

AD is an important medico-societal concern. Our current knowledge in the field supports that the dysregulation of the adenosinergic signaling is certainly important for the pathophysiological development of AD and constitutes a major therapeutic target that needs to be thoughtfully evaluated. Considering their known safety, evaluating caffeine and A_2A_R antagonists in clinical trials can be considered as a priority in the field. Whether modulating adenosine tone itself is a valuable strategy worth also to be investigated considering recent encouraging results (Lee et al., 2018), underlying mechanisms still need to be further elucidated. Owing to the important contribution of adenosine pathways to blood-brain-barrier permeability (Kim and Bynoe, 2015), physiological plasticity, neurotrophin actions (Rebola et al., 2008; Jerónimo-Santos et al., 2014) or even to the peripheral adaptive immune system lastly recognized as playing an important role in AD (Linden and Cekic, 2012; Antonioli et al., 2013; Laurent et al., 2018), careful investigations on processes underlying beneficial effects and potential side effects are warranted.

## Author Contributions

LC, KC, DB, CM, and VG-M wrote the manuscript. LC, KC, EF, AD, DV, LB, DB, CM, and VG-M reviewed the manuscript.

## Conflict of Interest Statement

The authors declare that the research was conducted in the absence of any commercial or financial relationships that could be construed as a potential conflict of interest.
